# Inducible T‐Cell Co‐Stimulator (ICOS) and ICOS Ligand: Dealing With a Two‐Faced Cancer Immunoregulatory System

**DOI:** 10.1002/cam4.71467

**Published:** 2025-12-31

**Authors:** Mina Nikanjam, Shumei Kato, Daisuke Nishizaki, Hirotaka Miyashita, Sarabjot Pabla, Mary K. Nesline, Heidi Ko, Jeffrey M. Conroy, Aung Naing, Razelle Kurzrock

**Affiliations:** ^1^ Division of Hematology‐Oncology University of California San Diego La Jolla California USA; ^2^ Department of Hematology and Oncology Dartmouth Cancer Center Lebanon New Hampshire USA; ^3^ OmniSeq (Labcorp) Inc. Buffalo New York USA; ^4^ Labcorp Oncology Durham North Carolina USA; ^5^ Department of Investigational Cancer Therapeutics MD Anderson Cancer Center Houston California USA; ^6^ Medical College of Wisconsin Cancer Center Milwaukee Wisconsin USA; ^7^ WIN Consortium Chevilly‐Larue France

**Keywords:** agonist, antagonist, ICOS, ICOS ligand, immune stimulation, immunosuppression

## Abstract

**Background:**

ICOS (inducible T‐cell co‐stimulator) and ICOS ligand (ICOSL) are part of an important, complex pathway that can lead to both immune stimulation and suppression. ICOS and ICOSL have heterogeneous expression patterns between and within tumor types.

**Methods:**

This review provides an overview of ICOS and ICOSL, their mechanisms of action, expression in cancer and other diseases, and clinical trials exploring therapies targeting ICOS.

**Results:**

Because of the bidirectional immune impact of the ICOS/ICOSL signaling pathway, both ICOS agonists and antagonists are under development and evaluation in clinical trials. The majority of clinical trials have focused on the development of ICOS agonists, with only one study exploring an ICOS antagonist; there have been no clinical trials developing ICOSL agonists or antagonists in oncology. ICOS can be expressed on immune‐activating effector T‐cell and immunosuppressive regulatory T‐cell (Tregs). Thus, it is critical to determine where and how ICOS is expressed in order to evaluate the role for agonists versus antagonists. To date, ICOS agonists have shown limited activity in patients with malignancies, perhaps because of the lack of biomarker‐based trials. However, an ICOS antagonist demonstrated a 44% response rate in angioimmunoblastic T‐cell lymphoma; ICOS is highly expressed on T‐follicular helper cells (type of CD4 cell) and proliferation of these cells may be a pathogenic mechanism for these lymphomas. A role for the ICOS/ICOSL signaling pathway has also been implicated outside of oncology, including in viral infections such as COVID‐19, and in autoimmune conditions such as asthma and systemic lupus erythematosus.

**Conclusion:**

Biomarker‐driven approaches will be important to individualize therapy and ascertain which cancer patients will derive the greatest benefit from ICOS‐directed combination therapy approaches.

## Graphical abstract

ICOS (inducible T‐cell co‐stimulator) and ICOS ligand (ICOSL) are part of an important, complex pathway that can lead to both immune stimulation and suppression. Both ICOS agonists and antagonists are under development as oncology therapeutics and biomarker driven approaches will be important to ascertain which patients will derive the greatest benefit these therapies.

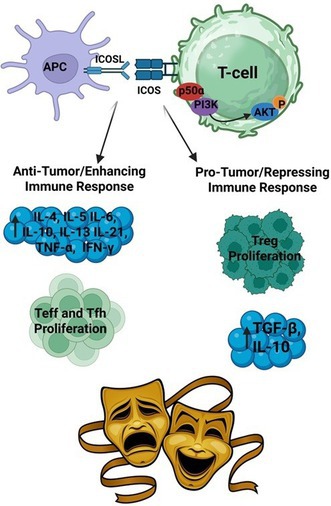

## 1 Introduction

While CTLA‐4 and PD‐1 inhibitors can lead to activation of the immune system and dramatic responses in some patients with immunologically driven tumors, there are many patients who fail to respond or eventually develop resistance to these therapies [1]. Some of these checkpoints are co‐expressed within a tumor and combining PD‐1 with CTLA‐4 inhibitors with each other or with LAG‐3 checkpoint blockade have been utilized as a method to overcome resistance and improve responses [2, 3, 4, 5, 6, 7, 8]. Exploiting additional novel immune checkpoints or immune stimulatory molecules with combination therapeutic approaches has the potential to provide additional benefit to immunomodulatory approaches.

ICOS (inducible T‐cell co‐stimulator; CD278) has a unique ligand (ICOSL) [9]. They form a complex and two‐faced immune signaling pathway which upon activation simultaneously leads to anti‐tumor responses/immune stimulation and immunosuppression [9, 10, 11, 12]. The interaction of ICOS and ICOSL can stimulate CD4 and CD8 ICOS‐positive T‐cell populations [11, 12], but also can induce proliferation of immunosuppressive regulatory T‐cells (Tregs) [10]. Many studies have focused on the ability of ICOS‐modulating agents to activate “cold tumors” and decrease Tregs, thus allowing T‐cell infiltration, which may create a tumor microenvironment more amenable to an anti‐tumor immune response [13]. Tumors with ICOS expression on tumor‐infiltrating CD4 and CD8 T cells may benefit from an ICOS agonist whereas tumors that express high levels of ICOS on Tregs may benefit from an ICOS inhibitor. A better understanding of ICOS and ICOSL expression patterns on different tumor types and in relation to other immune checkpoints can help establish the role for ICOS and ICOSL agonists and antagonists in targeted cancer therapy.

In the current review, we provide an overview of ICOS and ICOSL, their mechanisms of action, expression in cancer and other diseases, and clinical trials exploring therapies targeting ICOS.

## 2 Mechanism of Action of ICOS and ICOSL: Dealing With a Two‐Faced Immunoregulatory System

ICOS co‐stimulation can lead to multiple distinct processes during adaptive immune responses. These responses include but are not limited to induction, maintenance, and follicular homing of follicular helper T‐cells (Tfh) along with promoting thymus dependent antibody responses and driving antibody affinity maturation in the germinal cell reaction [14, 15, 16, 17, 18, 19, 20, 21, 22, 23, 24, 25, 26, 27, 28, 29, 30]. Naïve T cells do not express ICOS and ICOS expression is induced upon T cell activation. ICOSL is constitutively expressed by antigen‐presenting cells including B‐cells, macrophages, and dendritic cells, along with many somatic cells. In the peripheral blood, Tregs express the highest levels of ICOS [9].

### 2.1 Molecular Biology, Signal Transduction, and Functional Regulation of ICOS/ICOSL

ICOS is a 199‐amino acid protein which forms a 55–60 kDA sulfide linked homodimer. It is also known as CD278 and is a member of the costimulatory B7‐1/B7‐2–CD28/CTLA‐4 family [31]. ICOS has significant homology with co‐stimulatory CD28 and co‐inhibitor receptor CTLA‐4, which are T‐cell specific cell surface receptors that regulate the immune system [9, 32]. ICOS has a non‐redundant role to CD28 in T‐cell activation. ICOS lacks the MYPPPY motif present in CD28 and CTLA‐4 necessary for the interaction with CD80 and CD86 [32, 33]. The YMFM found in the cytoplasmic tail of ICOS recruits the p50alpha subunit of phosphatidylinositol 3‐kinase (PI3K). The cytoplasmic tail of ICOS has an intracellular signaling motif, IProx, which recruits TBK1, a member of the NF‐kB kinase family [34, 35].

**FIGURE 1:**
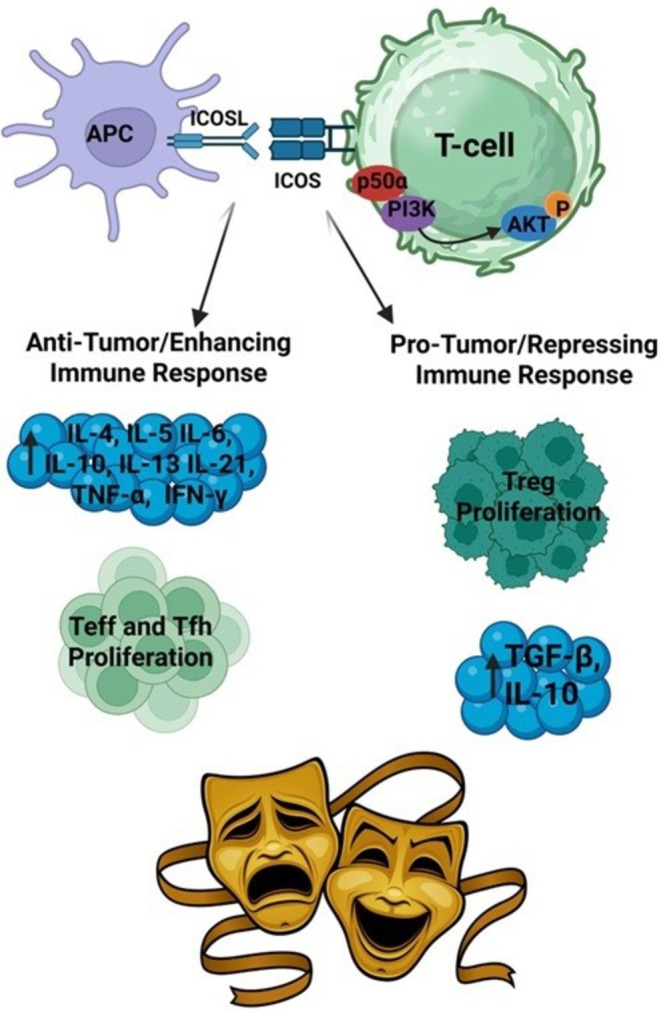
ICOS/ICOSL as a two‐faced immunoregulatory system. ICOS ligand on antigen‐presenting cells (APC) interacts with ICOS on T‐cells leading to activation of the PI3K/AKT pathway which increases interleukin production (IL‐4, IL‐5, IL‐6, IL‐10, IL‐13, IL‐21, IFN‐γ, TNF‐α) along with Teff and Tfh cell proliferation and survival. ICOS activation also leads to proliferation of regulatory T cells (Treg) cells and increased immunosuppressive cytokine (IL‐10, TGF‐β) production. Image created using biorender.com and Adobe Stock Image.

ICOS is found on T‐cells including CD4‐positive, CD8‐positive, and regulatory T‐cells [9] along with natural killer cells [36]. ICOSL is expressed on professional antigen‐presenting cells (APCs) including B‐cells, macrophages, and dendritic cells along with lung epithelium, myeloid‐derived suppressor cells [37], and certain endothelial cells [31, 38]. ICOSL primarily binds ICOS and is downregulated after interaction with ICOS. ICOSL can also bind other targets including integrin alphaVbeta3 integrin where it serves as an antagonist to maintain glomerular function in the kidney [39] and osteopontin which induces cell migration while inhibiting anchorage‐independent cell growth [40]. ICOSL on antigen‐presenting cells interacts with ICOS on T‐cells. The resulting ICOS co‐stimulation leads to cytokine production (IL‐4, IL‐5, IL‐6, IL‐10, IL‐13, and IL‐21) along with interferon gamma and TNF‐alpha [9, 41, 42], but does not lead to efficient IL‐2 production by activated T‐cells [9, 32]. This leads to effector T‐cell proliferation and survival through recruitment of phosphatidylinositol 3‐kinase (PI3K). The recruitment of the PI3K p50alpha regulatory subunit leads to robust AKT activation thereby promoting cell proliferation and survival [43] (Figure 1).

Concurrent binding of ICOSL on antigen‐presenting cells or tumor cells to ICOS leads to regulatory T‐cell proliferation and maintenance through secretion of IL‐10 and TGF‐beta. ICOS high Tregs can convert non‐T‐regs into IL‐10 expressing suppressive type‐1 regulatory T‐cells. Tregs suppress immune responses and help the tumor evade the immune response (Figure 1) [9, 31, 32, 34, 41, 42]. ICOSL triggering by ICOS also delivers a “reverse signal” modulating the activity of the ICOSL‐expressing cells including dendritic cells [44, 45, 46].

ICOS regulates effector T‐cell subset differentiation. It is necessary for the expansion of both Th1 and Th2 subsets. ICOS plays an important role in differentiation and activation of T cells into T follicular helper cells (Tfh) and migration of CXCR5 positive Tfh‐cells from the T‐B cell border into the follicle. Differentiation of the Th2 and Th17 subpopulations is dependent on ICOS and ICOS co‐stimulation can efficiently induce Th1 cytokine expression. ICOS‐mediated p85alpha recruitment promotes Tfh cell phenotype development. Simultaneous ICOS and CD3epsilon ligation leads to recruitment of TBK1, an inhibitor of the NF‐KB kinase family, which helps transition early Tfh cells to GC‐resident Tfh cells [9]. ICOS has been reported to help establish CD8+ tissue‐resident memory T‐cells and can regulate effector T‐cell subset differentiation [47]. Interrupting the intratumor CD8^+^ T cell and Treg crosstalk through inhibition of ICOS signaling prior to the administration of PD‐1 immunotherapy was previously shown to improve efficacy of PD‐1 directed therapy [48]. In contrast, ICOS inhibition or deficiency has also been reported to increase CD8+ T‐cell activity in studies of graft‐versus‐host disease, chronic viral infection, and cancer with sustained activation [49, 50]. Thus, the effect of ICOS on CD8+ T‐cell activity may be more complicated.

### 2.2 ICOS as a Therapeutic Target

ICOS agonists potentiated the effects of anti‐CTLA‐4 therapy in pre‐clinical studies and ICOS is upregulated with anti‐CTLA‐4 treatment. ICOS agonist monoclonal antibodies can help potentiate anti‐CTLA‐4 effects and may increase efficacy over administrating anti‐CTLA‐4 alone. ICOS‐high CD4+ and CD8+ T‐cell subpopulations have been suggested as a biomarker for clinical response [9]. ICOS‐targeting therapies are being studied in combination to anti‐CTLA‐4, PD‐1, or PD‐L1 antibodies (Table 1) [9, 51, 52, 53, 54, 55, 56, 57, 58].

**TABLE 1:** Examples of clinical trials targeting ICOS in cancer.^a^

Drug	Combination	Target	Phase	ICOS/ICOS ligand expression	Cancer type	Results/Comment	NCT number/References
ICOS agonists							
BMS‐986226	Ipilimumab (anti‐CTLA‐4 antibody) Nivolumab (anti‐PD‐1 antibody)	ICOS (agonist)	1/2	Not applicable	Solid tumors	Results not available Trial discontinued	NCT03251924
Feladilimab (GSK3359609)	Single agent, chemotherapy, pembrolizumab (anti‐PD‐1 antibody) Dostarlimab (anti‐PD‐L1 antibody) Cobolimab (anti‐TIM‐3 antibody) Bintrafusp alfa (anti‐PD‐L1 and TGF‐β bifunctional fusion protein)	ICOS (agonist)	1	Not applicable	Advanced solid tumors	Head and neck: Combination with pembrolizumab: ORR = 9/34 (26%) Urothelial expansion: Monotherapy: ORR 8% (1/13) Combination with pembrolizumab: ORR 22% (7/32)	NCT02723955 [51, 52]
	Immunotherapy	ICOS (agonist)	1	Not applicable	Solid tumors	Results not available	NCT03447314
	Pembrolizumab (anti‐PD‐1 antibody), 5FU‐platinum	ICOS (agonist)	2/3	Not applicable	Head and neck cancer	Results not available, trial on hold by sponsor	NCT04428333
	Tremelimumab (anti‐CTLA‐4 antibody) or standard of care (chemo or cetuximab)	ICOS (agonist)	1/2	Not applicable	Solid tumors	Results not available	NCT03693612
	Pembrolizumab (anti‐PD‐1 antibody)	ICOS (agonist)	2/3	Not applicable	Head and neck cancer	Results not available, trial on hold by sponsor	NCT04128696
Vopratelimab (JTX‐2011)	Anti‐PD‐1 or anti‐CTLA‐4	ICOS (agonist)	1/2	Not applicable	Refractory solid tumors	*N* = 201 enrolled Monotherapy: ORR = 1/70 (1.4%) Combination: ORR = 3/131 (2.3%) Comment: A potential genomic predictive biomarker of ICOS‐high CD4 T‐cell emergence was identified that demonstrated improvement in clinical outcomes, including OS (*p* = 0.0062); however, because this marker emerged with treatment, it cannot be used to select patients	NCT02904226 [53]
	JTX‐4014 (PD‐1 inhibitor)	ICOS (agonist)	2	RNA based tumor inflammation signature (TIS)	NSCLC	Combination: ORR = 9/33 (27.3%) Comment: The study was biomarker selected based on TIS^vopra^ score. TIS is an RNA based tumor inflammation signature of 18 genes associated with immune cell infiltration which was associated with ICOS high T‐cell emergence in the ICONIC study and predictive of response to anti‐PD‐1 therapy	NCT04549025 [54, 55]
	Anti‐PD‐1 or anti‐CTLA‐4	ICOS (agonist)	1/2	Not applicable	Solid tumors	Results not available	NCT04319224
	Anti‐CTLA‐4	ICOS (agonist)	2	Not applicable	NSCLC/urothelial cancer	Results not available	NCT03989362
XmAb23104	Single agent, ipilimumab (anti‐CTLA‐4 antibody)	ICOS (agonist)/PD‐1	1	Not applicable	Solid tumors	Monotherapy: ORR = 3/62 (4.8%)	NCT03752398 [56]
	XmAb22841 (CTLA‐4 and LAG3 bi‐specific antibody)	ICOS (agonist)/PD‐1	1/2	Not applicable	Melanoma	Results not available	NCT05695898
	Not applicable	ICOS (agonist)/PD‐1	2	Not applicable	Advanced sarcoma	Results not available	NCT05879185
ICOS antagonist							
MEDI‐570	Not applicable	ICOS (antagonist)	1	Not applicable	Peripheral T‐cell lymphoma follicular variant and angioimmunoblastic T‐cell lymphoma	ORR = 7/23 (30%) ORR in angioimmunoblastic T‐cell lymphoma was 44%	NCT02520791 [57]
ICOS agonist and depleter							
KY1044/Alomfilimab SAR445256	Single agent, Atezolizumab (anti‐PD‐L1 antibody)	ICOS (stimulates Teff with low ICOS/depletes Treg with high ICOS)	1/2	Not applicable	Advanced cancer	ORR = 5/103 (5%)	NCT03829501 [58]

Abbreviations: ICOS, inducible T‐cell co‐stimulator; ORR, overall response rate; Teff, effector T‐cell; Treg, regulatory T‐cell.

^a^ Updated as of July 13, 2024.

Given that ICOS/ICOSL pathway activation can both stimulate and inhibit the immune system, developing appropriate targets and therapeutics can be challenging. Tumors in which ICOS is mainly expressed on Tregs may be better targets for antagonists whereas those with expression by inflammatory or cytotoxic T cells may prove better targets for agonists; thus, an ICOS‐positive effector T‐cell (Teff)/Treg ratio may be of benefit in determining the value of an agonist versus antagonist [31]. Finally, in diseases such as T‐cell lymphoma, attenuating the effects of ICOS stimulation may be important and, therefore, an ICOS antagonist may be appropriate in this situation [59].

## 3 Expression Patterns of ICOS and ICOSL in Cancer

ICOS/ICOSL pathway activation has been observed in multiple cancer types including melanoma [60], multiple myeloma [61], breast [62], ovarian [63], gastric [64], liver [65], Merkel cell carcinoma [66], angioimmunoblastic T‐cell lymphoma [67], and colorectal cancers [68]. ICOSL expression has also been seen in Hodgkin lymphoma [69], B‐cell lymphoma [70], acute myeloid leukemia [71], glioblastoma multiforme [72], and rhabdomyosarcoma [73]; however its exact role in these cancers is still under investigation. ICOSL expression has been detected on tumor cells for melanoma, multiple myeloma, colorectal cancer, B‐cell lymphoma, acute myeloid leukemia, glioblastoma multiforme, and rhabdomyosarcoma, whereas it was noted to be present on immune cells infiltrating the tumors for breast, ovarian, gastric, and liver cancers. ICOS expression was detected on tumor cells for Merkel cell carcinoma and angioimmunoblastic T‐cell lymphoma [60, 61, 62, 63, 64, 65, 66, 67, 68, 70, 71, 72, 73].

ICOS expression on Tregs after IL‐2 therapy and the presence of Tregs in tumor infiltrating lymphocytes (TILs) has been associated with poor clinical outcome in metastatic melanoma [74, 75], however ICOS presence on other TILs has been associated with better prognosis [76]. ICOS‐high Tregs were previously observed to have superior immunosuppressive activity in melanoma than ICOS‐low Tregs [75]. The presence of CD4+ ICOS+T‐cells in the tumor microenvironment and peripheral blood was associated with a good treatment response to ipilimumab [77]. Elevated ICOS‐positive Tregs in localized renal cell carcinoma were associated with poor prognosis [78]. ICOS expression on Tregs in breast cancer also correlated with poor prognosis [79]. ICOS was elevated on Tregs of ovarian cancer patients [80] at higher levels than in melanoma. ICOSL on plasmacytoid dendritic cells [63] was felt to enhance the immunosuppressive activity of Tregs. A higher proportion of Tregs expressing ICOS were found in later stage gastric cancer [64]. In colorectal cancer, ICOS was mainly expressed by CD8+ and CD4+ T‐cells; thus a higher percentage of ICOS+ T‐cells in the peripheral blood and tumor was associated with improved prognosis [68]. ICOS was also associated with higher CTLA‐4 and PD‐1 expression on lymphocytes in colorectal cancer [68].

Increased ICOS expression on CD4+ T‐cells of patients treated with anti‐CTLA‐4 therapies has been seen in non‐small cell lung cancer [81], bladder cancer [82], and breast cancer [83]. ICOS expression was higher on CD4+ T‐cells following ipilimumab compared to gp100 DNA vaccine in melanoma patients [84]. Combining ipilimumab and liver radiation also led to increased peripheral T‐cell ICOS expression in a prior phase I study [85]. ICOS was also shown to be an important element in the persistence of CD4+ chimeric Ag receptor (CAR) T cells, which have been approved for several hematological malignancies and are in clinical trials for a wide variety of other malignancies [86].

Miyashita et al. explored the relationship between ICOS and ICOSL expression with microsatellite instability high (MSI‐H), tumor mutational burden (TMB), and PD‐L1 expression and found that PD‐L1 ≥ 1% was associated with significantly higher ICOS expression [87]. A recent study evaluated ICOS and ICOSL RNA expression in 514 cancers. ICOS and ICOSL varied between and within tumor types. High ICOS expression was present in 14% of cancers and was independently associated with high PD‐1, PD‐L1, and CTLA‐4 expression [88].

These studies suggest that ICOS/ICOSL are heterogeneously expressed across a variety of cancers. However, variability of ICOS/ICOSL expression has been noted between and within tumor types and between cell types as well. The ICOS/ICOSL system may also be modulated by administration of various immunotherapy agents, and the complexity of expression patterns may explain why ICOS has been associated with both better and worse prognosis, depending on the tumor type and assays used.

## 4 Clinical Trials Targeting ICOS

The interaction of ICOS and ICOSL can stimulate CD4 and CD8 ICOS‐positive T‐cell populations, but also can induce proliferation of immunosuppressive regulatory T‐cells (Tregs). Thus, ICOS agonists and antagonists are both under development as novel therapies (Table 1) [51, 52, 53, 54, 55, 56, 57, 58]. The majority of clinical trials to date have focused on the development of ICOS agonists, with only one study exploring an ICOS antagonist (MEDI‐570). There have been no clinical trials developing ICOSL agonists or antagonists in oncology, but such trials exist outside of oncology with, for instance, development of an anti‐ICOSL antibody for systemic lupus erythematosus (SLE).

### 4.1 Rationale and Application of ICOS Agonist Activity in Oncology

Several clinical trials have evaluated ICOS agonists alone or together with other agents such as checkpoint inhibitors (Table 1). For the most part, these trials show modest/disappointing activity. Biomarkers for patient selection have mostly not been addressed and, in the trials in which such biomarkers have been examined, results remain unclear, probably in large part because of the complexity and heterogeneity of the ICOS signaling pathway.

JTX2011/vopratelimab is an ICOS agonist which was evaluated in a phase I/II trial for refractory solid tumors either alone, in combination with anti‐PD‐1, or in combination with anti‐CTLA‐4 therapy. A total of 201 subjects were enrolled in the study. The overall response rate was 1.4% for monotherapy and 2.3% for combination therapy. While ICOS‐high CD4 T‐cell emergence was noted to improve clinical outcomes including overall survival, it was not used as a biomarker to select patients as it was upregulated with treatment. In the combination therapy arm, 11.5% of patients experienced grade 3–4 treatment‐related adverse events with the most common events being elevated aspartate aminotransferase (AST) (3.1%), hyponatremia (1.5%), maculopapular rash (0.8%), and abdominal pain (0.8%) (NCT02904226; ICONIC) [53]. JTX2011/vopratelimab was also evaluated in a phase 2 study with the PD‐1 inhibitor JTX‐4014 in non‐small cell lung cancer following one prior platinum‐containing regimen (SELECT study). The study used a biomarker‐selected approach based on TIS^vopra^ score. TIS is an RNA based tumor inflammation signature of 18 genes associated with immune cell infiltration which was associated with ICOS high T‐cell emergence following treatment in the ICONIC study and predictive of response to anti‐PD‐1 therapy. TIS^vopra^ positivity was based on a specific threshold of TIS determined by the sponsor (NCT04549025) [54]. The overall response rate for combination therapy was 27.3% (9/33 subjects). Most adverse events were mild or moderate with few treatment related serious adverse events [55]. JTX2011/vopratelimab is also being investigated in a phase I/II study of solid tumors in combination with either anti‐PD‐1 or anti‐CTLA‐4 therapy (NCT04319224) and in combination anti‐CTLA‐4 therapy in a phase 2 study of non‐small cell lung cancer and urothelial cancers (NCT03989362). Results were not available for these studies.

GSK3359609/feladilimab is an ICOS agonist. It was evaluated as a single agent along with in combination with multiple other therapies chemotherapy, pembrolizumab (anti‐PD‐1 antibody), dostarlimab (anti‐PD‐L1 antibody), cobolimab (anti‐TIM‐3 antibody), bintrafusp alfa (PD‐L1 and TGF‐β bifunctional fusion protein) in a phase 1 study of advanced solid tumors (INDUCE‐1). Preliminary results in combination with pembrolizumab in a treatment‐naïve head and neck squamous cell carcinoma cohort had an overall response rate of 26%. Less than 10% of patients had grade 3 or greater treatment‐related adverse events (NCT02723955) [51]. In the urothelial expansion cohort, monotherapy had an 8% overall response rate while combination with pembrolizumab increased to 22%. Grade 3 or greater treatment‐related side effects were present in 9% of patients in the combination therapy arm [52]. It is also being investigated in combination with immunotherapy in a phase 1 study for solid tumors (NCT03447314), in combination with pembrolizumab/5‐fluorouracil/platinum in a phase II/III study of head and neck cancer (NCT04428333; INDUCE‐4), in combination with pembrolizumab in a phase II/III study of PD‐1 positive head and neck cancer (NCT04128696; INDUCE‐3), and a phase I/II study of solid tumors in combination with tremelimumab (anti‐CTLA‐4 antibody) or standard of care (paclitaxel, docetaxel, or cetuximab) (NCT03693612). Results from these trials are not available and enrollment for INDUCE‐3 and INDUCE‐4 have been put on hold by the sponsor.

XmAb23104 is a bispecific antibody which serves both as an ICOS agonist and a PD‐1 inhibitor. It has been explored as a single agent and in combination with ipilimumab (anti‐CTLA‐4 antibody) in a phase 1 study of solid tumors. In the single agent arm, ORR was 4.8%. Grade 3 or higher treatment‐related adverse events were present in 6 subjects (9.7%) with two Grade 3–4 immunotherapy‐related adverse events (Grade 3 pruritis and asymptomatic Grade 4 lipase elevation) (NCT03752398) [56]. It is also being investigated in a phase I/II trial in melanoma in combination of with XmAb22841 a bispecific antibody targeting CTLA‐4 and LAG3 (NCT05695898) along with a phase 2 trial of advanced sarcoma as a single agent (NCT05879185). These trials are ongoing.

BMS‐986226 is an ICOS agonist which was being evaluated alone or combination with ipilimumab (anti‐CTLA‐4 antibody) or nivolumab (anti‐PD‐1 antibody) in a phase I/II study of solid tumors. The study was discontinued by the sponsor and does not have available results (NCT03251924).

Anti‐ICOS antibodies can also lead to depletion of ICOS‐expressing cells. KY1044/alomfilimab SAR445256 is a dual mechanism molecule which binds to ICOS with high affinity. It stimulates Teff cells with low ICOS and preferentially depletes Treg cells with high ICOS. KY1044/alomfilimab evaluated in a phase 1 study of advanced cancers and found to have a 5% response rate. Grade 3 or greater treatment‐related adverse events occurred in < 8% of patients (NCT02520791) [58].

### 4.2 Rationale and Application of ICOS Antagonist Activity in Oncology

There are two major settings in which ICOS antagonists may have activity. First, ICOS is highly expressed on T‐follicular helper cells (type of CD4 cell) and proliferation of these cells is postulated as a pathogenic mechanism for certain T‐cell lymphomas [57]. A second setting may be to counteract ICOS expression on Tregs, where ICOS signaling promotes the generation and immunosuppressive ability of the Treg cells [9].

MEDI‐570 is an ICOS antagonist being evaluated in a phase 1 study of peripheral T‐Cell lymphoma follicular variant and angioimmunoblastic T‐cell lymphoma. The response rate in angioimmunoblastic T‐cell lymphoma was 44%. No dose‐limiting toxicities were reported in the dose escalation phase. There were no drug discontinuations due to adverse events (NCT02520791) [57].

### 4.3 Challenges and Future Directions for Clinical Trials

Given the dual nature of ICOS in immune stimulation and inhibition it will be critical to determine which tumor types would benefit from an ICOS agonist versus antagonist. The nature of the system may lend to biomarker driven clinical trials to select the optimal patients for each therapy. With the low activity of single agents, determining the ideal pairing and timing of ICOS‐targeted therapies with PD‐1, CTLA‐4, and other checkpoint inhibitors will be critical. While one could simultaneously administer combination therapies with an ICOS pathway affecting agent and a distinct immune checkpoint inhibitor, there may be benefit to a staggered starting approach. There are no clinical trials of ICOSL agonists or antagonists in oncology thus exploring the role for these therapeutics in combination oncology therapy will be of interest for future clinical trials.

Additional exploration into ICOS and ICOSL expression patterns and correlation to response would be beneficial for biomarker selected trials. It may be that ICOSL agonists are most effective in the presence of high ICOS and low ICOSL. ICOS agonists might have greatest efficacy when ICOS is low. The cells on which ICOS and ICOSL are expressed likely play a significant role. Clinical trials where biomarkers are assessed prior to the start of therapy and throughout therapy to determine which expression patterns benefit the most from ICOS directed therapies would be of great benefit. Additional exploration into the distinct roles of ICOS signaling in CD4 and CD8 T cells will also be of importance to determine benefit for ICOS directed therapies based on cell expression given the suggestion that they may have opposite effects [49].

## 5 ICOS/ICOS Ligand Beyond Oncology

ICOS/ICOSL has been explored outside of oncology, including in viral infections, and in autoimmune conditions such as asthma and systemic lupus erythematosus (SLE). A study of COVID‐19 in a non‐small cell lung cancer cohort found that high ICOS expression correlated with high ACE2 expression in normal lung tissue (ACE2 being the receptor that permits COVID‐19 cellular penetration) and suggested that high ICOS expression may predispose to the inflammatory/cytokine over‐reaction that causes serious COVID‐19 pneumonia [89]. ICOS‐positive CD4+ T cells have also been found to correlate with the development of protective antibody responses by memory B cells after influenza vaccination [90]. ICOS is a marker for circulating T‐follicular helper cells, which induce viral‐specific memory B cells to differentiate into plasma cells leading to protective antibody response in viral infection [34]. Homozygous ICOS deficiency can result in common variable immunodeficiency (CVID) which predisposes patients to frequent bacterial infections [91].

ICOS had been thought to play a role in abnormal T‐cell activation in SLE [92]. The anti‐ICOSL antibody AMG 557 has been studied in SLE. A phase I clinical trial administer AMG557 subcutaneously with an intradermal injection of the neoantigen keyhole limpet hemocyanin (KLH) in patients with mild, stable SLE and found it effective in diminishing isotype‐switched antibody production, but not IgM production [93]. Additional data from a phase IB study of AMG557 in mild, stable SLE patients demonstrated an acceptable safety profile and was effective reducing the anti‐KLH IgG response [94]. AMG557 was overall felt to be safe and have biologic activity relevant to SLE [95].

ICOS/ICOSL signaling was found to have a role in induction of ILC2‐mediated cytokine production which can lead to airway hyperreactivity, which is an indicator of asthma. Anti‐ICOS antibody treatment decreased IL‐33 induced airway hyperreactivity in a pre‐clinical model [96].

In the graft rejection field, the combination of anti‐ICOS with anti‐CTLA4 antibody therapy prevented cardiac allograft rejection in pre‐clinical studies in rats [97, 98]. However, anti‐ICOS and anti‐CTLA4 combined therapy was not effective at preventing graft rejection after kidney transplant in non‐human primates [99].

## 6 Conclusions

A variety of immunoregulatory molecules are being explored in order to improve outcomes and overcome resistance to PD‐1/CTLA‐4/LAG‐3 checkpoint inhibition in cancer. ICOS and ICOSL are part of an important and complex pathway, which can lead to both immune stimulation and suppression. ICOS agonists, ICOS antagonists, and ICOSL antagonists have been explored as therapeutics for both oncology and non‐oncology applications (Table 2). Single‐agent ICOS modulating agents are felt to have reasonable safety profiles but limited efficacy in oncology; thus future studies focus on combination approaches with anti‐PD‐1/PD‐L1 or anti‐CTLA‐4 therapeutics. Exploration of sophisticated biomarkers looking at endogenous ICOS/ICOSL expression by cell type and in individual tumors may also be necessary. Indeed, given that ICOS can be expressed on effector T‐cells and on regulatory T‐cells, it will be critical to determine which cell type in an individual tumor expresses ICOS/ICOSL in order to ascertain the role for an agonist versus antagonist. Tumors with ICOS mainly on inflammatory/cytotoxic T cells with minimal Treg infiltration or minimal ICOS expression on Tregs may prove better targets for agonists. In contrast, tumors with high levels of ICOS on Tregs with minimal ICOS expression on inflammatory/cytotoxic T cells or high levels of Treg infiltration may be better served with an ICOS antagonist. Of interest, an ICOS antagonist was found to have a 44% response rate in angioimmunoblastic T‐cell lymphoma [57]. In conclusion, the ICOS/ICOSL pathway represents a novel immune checkpoint with potential for improving outcomes in combination cancer immunotherapy approaches.

**TABLE 2:** Examples of ICOS/ICOSL modulating agents and applications in oncology and other diseases.

	Possible applications	References
ICOS agonists	Oncology: NSCLC, head and neck cancers, solid tumors	[51, 52, 53, 54]
ICOS antagonists	Oncology: Angioimmunoblastic T‐cell lymphoma Non‐oncology: Asthma Transplant rejection	[57, 96, 97, 98]
ICOSL antagonist	SLE	[94, 95]

Abbreviations: ICOS, inducible T‐cell co‐stimulator; ICOSL, inducible T‐cell co‐stimulator ligand; NSCLC, non‐small cell lung cancer; SLE, systemic lupus erythematosus.

## Author Contributions

**Mina Nikanjam:** conceptualization (equal), writing – original draft (equal), writing – review and editing (equal). **Shumei Kato:** writing – review and editing (equal). **Daisuke Nishizaki:** writing – review and editing (equal). **Hirotaka Miyashita:** writing – review and editing (equal). **Sarabjot Pabla:** writing – review and editing (equal). **Mary K. Nesline:** writing – review and editing (equal). **Heidi Ko:** writing – review and editing (equal). **Jeffrey M. Conroy:** writing – review and editing (equal). **Aung Naing:** writing – review and editing (equal). **Razelle Kurzrock:** conceptualization (equal), writing – original draft (equal), writing – review and editing (equal).

## Funding

This work was supported by the National Institutes of Health (Grants 5U01CA180888‐08 and 5UG1CA233198‐05).

## Conflicts of Interest

Dr. Kurzrock has received research funding from Boehringer Ingelheim, Debiopharm, Foundation Medicine, Genentech, Grifols, Guardant Incyte, Konica Minolta, Medimmune, Merck Serono, OmniSeq, Pfizer, Sequenom, Takeda, and TopAlliance and from the NCI; as well as consultant and/or speaker fees and/or advisory board/consultant for Actuate Therapeutics, AstraZeneca, Bicara Therapeutics Inc., Biological Dynamics, Caris, Datar Cancer Genetics, Daiichi, EISAI, EOM Pharmaceuticals, Iylon, LabCorp, Merck, NeoGenomics, Neomed, Pfizer, Precirix, Prosperdtx, Regeneron, Roche, TD2/Volastra, Turning Point Therapeutics, X‐Biotech; has an equity interest in CureMatch Inc. and IDbyDNA; serves on the Board of CureMatch and CureMetrix, and is a co‐founder of CureMatch. Dr. Kato serves as a consultant for Medpace, Foundation Medicine, NeoGenomics and CureMatch. He receives speaker's fee from Chugai, Roche/Genentech and Bayer, and advisory board for Pfizer. He has research funding from ACT Genomics, Sysmex, Konica Minolta, OmniSeq, Personalis and Function Oncology.

## Data Availability

Data sharing not applicable to this article as no datasets were generated or analyzed during the current study.
